# microRNA‐Mediated Regulation of Oxidative Stress in Cardiovascular Diseases

**DOI:** 10.1002/jcla.70017

**Published:** 2025-04-04

**Authors:** Sakhavat Abolhasani, Yasin Ahmadi, Davood Fattahi, Yavar Rostami, Khalil Maleki Chollou

**Affiliations:** ^1^ Department of Basic Sciences and Health Sarab Faculty of Medical Sciences Sarab East Azerbaijan Iran; ^2^ Department of Medical Laboratory Science Komar University of Science and Technology Sulaymaniyah Kurdistan Region Iraq; ^3^ School of Pharmacy and Biomolecular Sciences Liverpool John Moores University Liverpool UK

**Keywords:** cardiovascular diseases, microRNAs, oxidative stress, reactive oxygen species, theranoMiRNAs

## Abstract

**Background:**

Cardiovascular diseases (CVDs) are the leading cause of mortality globally, often linked to oxidative stress. MicroRNAs (miRNAs) have emerged as significant regulators of oxidative stress within the cardiovascular system.

**Objective:**

This review examines the complex relationship between miRNAs and oxidative stress, clarifying their effects on gene expression pathways related to ROS production and detoxification in CVDs.

**Methods:**

From August to October 2024, we conducted a comprehensive search of PubMed, Scopus, Web of Science, and Google Scholar for studies published between 2014 and 2024 investigating the role of miRNAs in oxidative stress and cardiovascular diseases.

**Results:**

Specific miRNAs have been identified as critical regulators in the pathophysiology of CVDs, with distinct expression patterns correlated with conditions such as hypertension, coronary artery disease, and heart failure. For instance, miR‐21 exacerbates oxidative stress by targeting genes essential for redox homeostasis, while miR‐210 promotes endothelial cell survival under hypoxic conditions by mitigating ROS levels.

**Conclusion:**

The reciprocal relationship between miRNAs and oxidative stress highlights the potential for therapeutic interventions targeting miRNA expression and activity in managing CVDs. Understanding these molecular mechanisms is vital for developing innovative strategies to address oxidative damage in cardiac tissues and improve cardiovascular health outcomes.

## Graphical abstract

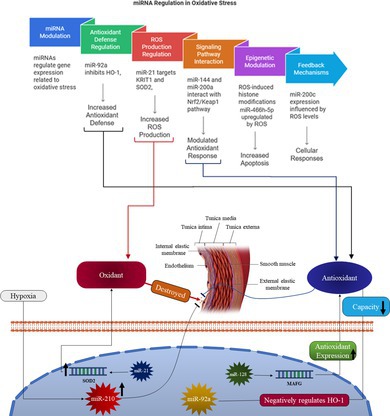

## 1 Introduction

Cardiovascular diseases (CVDs) are the leading cause of global mortality, often resulting in sudden deaths with undetermined causes [1]. While established clinical risk factors contribute significantly to this concerning trend, research in the emerging field of microRNAs (miRNAs) has revealed their fundamental involvement in the pathophysiology of various cardiovascular conditions, including coronary artery disease, hypertension, stroke, and heart failure [2, 3, 4]. miRNAs are small, non‐coding RNA molecules, typically around 22 nucleotides in length, that regulate gene expression by binding to the 3′ untranslated regions of target messenger RNAs (mRNAs). This binding can repress translation or degrade mRNA, thereby affecting numerous biological processes such as cell proliferation, apoptosis, differentiation, and responses to oxidative stress [5, 6].

The heart, characterized by its high metabolic demands and limited regenerative capacity, is especially susceptible to oxidative stress—a condition further exacerbated by diseases that increase the production of reactive oxygen species (ROS) [7]. This oxidative stress can lead to cardiomyocyte hypertrophy and DNA damage. Although endogenous scavenging enzymes and antioxidants contribute to the moderation of oxidative stress, the complex molecular mechanisms regulating these responses in cardiac tissue remain poorly understood [8]. Deterioration in cardiac function can significantly worsen conditions like congestive heart failure, prompting researchers to investigate novel molecular targets for therapeutic intervention [9].

Recent studies have highlighted a complex interaction between miRNAs and oxidative stress within the context of CVDs [10, 11]. Certain miRNAs have been identified as regulators of gene expression related to oxidative stress responses, while elevated oxidative stress can also modify miRNA expression profiles [11, 12]. Besides, specific miRNAs have been demonstrated to enhance antioxidant defenses by targeting genes involved in ROS production and detoxification pathways [13]. Conversely, increased oxidative stress can disrupt the expression levels of miRNAs essential for maintaining cellular homeostasis and functionality [12]. This bidirectional relationship indicates that therapeutic strategies aimed at modulating miRNA pathways may offer promising approaches for alleviating oxidative stress‐related damage in CVDs [14].

Investigating miRNAs concerning oxidative stress represents a promising pathway for enhancing our understanding of CVDs. By analyzing the complex roles of these small RNA molecules in regulating oxidative stress responses, we may identify potential biomarkers for early diagnosis and novel therapeutic targets expected to reduce oxidative damage to cardiac tissue [15]. This review aims to elucidate the essential roles of miRNAs in the onset and progression of CVDs through their regulatory effects on oxidative stress.

## 2 Materials and Methods

This narrative review was conducted to explore the regulatory roles of miRNAs in oxidative stress pathways within the context of cardiovascular diseases (CVDs). To ensure a comprehensive and systematic approach, the following methodology was employed.

### 2.1 Literature Search Strategy

A structured literature search was conducted across multiple electronic databases, including PubMed, Scopus, Web of Science, and Google Scholar, between August 2024 and October 2024. The search strategy incorporated a combination of keywords, including “microRNAs,” “oxidative stress,” “cardiovascular diseases,” “reactive oxygen species (ROS),” “miRNA regulation,” “therapeutic miRNAs” and “theranoMiRNAs”.

Notably, the search results for miRNAs and their correlation with cardiovascular disease and oxidative stress were required to include the terms “microRNAs,” “oxidative stress,” and “cardiovascular diseases,” as these were considered essential criteria. Additional keywords, such as “reactive oxygen species (ROS),” “miRNA regulation,” and “therapeutic miRNAs,” were included to refine the search but were not mandatory for article selection.

The predicted target genes of various miRNAs, particularly those presented in Section 3.3, “Differential Expression of miRNAs in CVDs,” including miR‐210, miR‐21, the miR‐200 family, miR‐128, and miR‐92a, play crucial roles in the regulation of oxidative stress and are implicated in the pathogenesis or prevention of cardiovascular diseases. The miRNA target prediction data for these miRNAs were extracted from miRDB (http://mirdb.org), a reliable online database that provides miRNA‐target interaction predictions based on machine learning algorithms. Detailed data on these predicted target genes is provided as Appendix S1 and is available upon request.

### 2.2 Eligibility Criteria

Studies were included if they were published in peer‐reviewed journals between 2014 and 2024 and focused on the role of miRNAs in oxidative stress and cardiovascular diseases. Eligible articles encompassed original research studies, systematic reviews, meta‐analyses, and review articles written in English. Studies were excluded if they did not specifically address oxidative stress or cardiovascular diseases, were not published in peer‐reviewed journals, or consisted of conference abstracts or unpublished data. Additionally, research focusing exclusively on non‐human models without direct translational relevance was not considered.

### 2.3 Data Extraction

Key information from the selected studies was systematically extracted and organized in a standardized table format. The extracted data included study type, categorized as experimental, clinical, or computational, along with the specific miRNAs investigated and their roles in oxidative stress pathways. Additionally, mechanistic insights into miRNA‐target interactions were documented to elucidate their regulatory functions. The relevance of these findings to cardiovascular disease pathophysiology and their potential therapeutic applications were also analyzed to provide a comprehensive understanding of the subject.

### 2.4 Quality Assessment

To ensure scientific rigor, we prioritized high‐impact studies published in reputable journals. Additionally, recent advancements such as computational validation tools (e.g., TargetScan, miRDB) were integrated into our analysis to strengthen the validity of reported findings (Appendix S1).

## 3 Results

### 3.1 Mechanisms of Oxidative Stress in CVDs

Oxidative stress, characterized by an imbalance between reactive oxygen species (ROS) and antioxidants, is a key factor in the pathogenesis of CVDs [16].

#### 3.1.1 Sources of ROS

ROS arise from multiple sources within the cardiovascular system, significantly contributing to oxidative stress and the development of CVDs [17]. ROS in the cardiovascular system originates from various sources. Mitochondria are primary producers during aerobic respiration, with dysfunction under pathological conditions like ischemia leading to excessive ROS and cellular damage [18]. NADPH oxidases (NOX), particularly NOX2 and NOX4, are major contributors in vascular cells, generating superoxide anions. Elevated NOX activity is linked to diseases such as hypertension and atherosclerosis [19, 20]. Other enzymatic sources, including xanthine oxidase and uncoupled nitric oxide synthase, further amplify oxidative stress by producing hydrogen peroxide and reactive metabolites [21]. The interaction among these sources creates a detrimental cycle; elevated ROS levels lead to increased oxidative damage, thereby worsening cardiovascular dysfunction [22].

#### 3.1.2 Impact of ROS on Cardiovascular Function

Reactive oxygen species (ROS) profoundly impact cardiovascular function by disrupting cellular signaling and compromising structural integrity [23]. Elevated ROS levels contribute to endothelial dysfunction by impairing nitric oxide (NO) signaling, leading to reduced vasodilation and increased vascular stiffness, which are precursors to atherosclerosis [24]. In cardiomyocytes, excessive ROS triggers lipid peroxidation, DNA damage, and mitochondrial dysfunction, resulting in cell death, particularly in heart failure and ischemia‐reperfusion injuries [25]. Furthermore, ROS promotes vascular inflammation by activating inflammatory pathways and attracting immune cells [26], accelerating the progression of atherosclerosis and other cardiovascular diseases [27].

Chronic oxidative stress drives cardiac remodeling, marked by fibrosis and hypertrophy, as ROS stimulate fibroblast proliferation and collagen deposition, leading to impaired cardiac function [28, 29]. The various sources of ROS in cardiovascular disease significantly contribute to oxidative stress, negatively affecting cardiovascular health through mechanisms like endothelial dysfunction, cardiomyocyte injury, inflammation, and structural remodeling. A comprehensive understanding of these mechanisms is crucial for the development of targeted therapeutic strategies aimed at alleviating oxidative stress in CVDs [30].

### 3.2 Role of miRNAs in Regulating Oxidative Stress

miRNAs are small, non‐coding RNA molecules that play a crucial role in the regulation of gene expression and have recently been recognized as essential regulators of oxidative stress [31]. They influence various cellular processes, particularly those associated with responses to oxidative stress, a condition implicated in several diseases, including neurodegenerative disorders, CVDs, and cancer [32].

#### 3.2.1 Mechanistic Insights of miRNAs in Oxidative Stress Pathways

In the context of CVDs and other oxidative stress‐related conditions, miRNAs modulate the expression of genes involved in both reactive oxygen species (ROS) production and detoxification [33]. This section discusses specific miRNAs associated with the regulation of oxidative stress pathways and provides insights into their mechanisms of action (Table 1).

**TABLE 1:** The interactions between specific miRNAs and oxidative stress mechanisms emphasize their implications for cardiovascular health.

Category	microRNA	Target gene(s)	Mechanism	Effect on oxidative stress
Modulation of antioxidant defense	miR‐92a	Heme oxygenase‐1 (HO‐1)	Inhibits HO‐1, a key enzyme that produces antioxidant biliverdin and bilirubin	Decreased miR‐92a → increased HO‐1 → enhanced antioxidant capacity → reduced oxidative stress [34]
Regulation of ROS‐producing enzymes	miR‐21	Targets and downregulates genes critical for redox homeostasis	Targets and downregulates genes critical for redox homeostasis	Increased miR‐21 → decreased KRIT1 & SOD2 → increased ROS accumulation [35]
Key signaling pathways	miR‐144	Nrf2	Targets Nrf2 or its upstream regulators, influencing antioxidant gene expression	Modulates Nrf2 pathway → affects antioxidant response to oxidative stress [36]
	miR‐200a	Nrf2	Similar to miR‐144, it targets Nrf2 or its regulators	Modulates Nrf2 pathway → affects antioxidant response to oxidative stress [36]
Epigenetic modulation	miR‐466 h‐5p	Anti‐apoptotic genes	Upregulated by ROS via inhibition of histone deacetylases (HDACs)	Increased miR‐466h‐5p → increased apoptosis through targeting anti‐apoptotic genes [37]
Feedback mechanisms	miR‐200c	Not specified	Induced by elevated ROS levels, influences cellular responses to oxidative stress	Suggests reciprocal relationships; targeting may provide therapeutic paths for CVDs [38]
Regulation of mitochondrial function	miR‐155	SIRT1, PGC1‐α	Target genes involved in mitochondrial biogenesis and function	Increased miR‐155 → inhibition of mitochondrial function → enhanced oxidative stress [39]
Regulation of inflammation	miR‐146a	IRAK1, TRAF6	Modulates inflammatory responses and can influence oxidative stress	Increased miR‐146a → downregulation of inflammatory pathways → decreased ROS production [40]
	miR‐34a	CDK6, Bcl2	Influences oxidative stress and apoptosis pathways	Increased miR‐34a → enhanced apoptosis → can result in increased oxidative stress [41]
Modulation of apoptosis	miR‐30	BNIP3, Beclin‐1	Regulates autophagic processes and apoptosis, protective against oxidative stress	Increased miR‐30 → enhanced autophagy → reduced oxidative stress [41]
Mitochondrial regulation	miR‐125b	Bcl‐2, MDM2	Regulates cell survival pathways impacting mitochondrial function	Increased miR‐125b → increased apoptosis → reduced mitochondrial function & ROS levels [42]
Regulation of cell proliferation	miR‐135	EGFR, PTEN	Modulates signaling pathways affecting cell proliferation and survival	Increased miR‐135 → altered cell growth → increased oxidative stress in certain contexts [43]
involvement in angiogenesis	miR‐126	SPRED1, VEGF	Implicated in endothelial protection and angiogenesis	Increased miR‐126 → enhanced angiogenic factors → reduced ischemic oxidative stress [44]
Regulation of cardiac hypertrophy	miR‐208a	Myosin heavy chain	Promotes the hypertrophic response in cardiomyocytes	Increased miR‐208a → cardiac remodeling → may enhance oxidative stress in hypertrophy [45]
Protection against ischemia	miR‐1	CAV1, SIRT1	Enhances cardiomyocyte survival during ischemic conditions	Increased miR‐1 → protection against cell death → ameliorates oxidative stress [46]
Regulation of lipid metabolism	miR‐27a	PPARγ, ABCA1	Implicated in lipid metabolism and may impact oxidative stress levels	Increased miR‐27a → altered lipid profile → may affect inflammatory and oxidative stress [47]
Regulation of endothelial function	miR‐126	SPRED1, VEGF	Protects endothelial cells and promotes angiogenesis	Increased miR‐126 → enhanced vascular function → reduced oxidative stress in endothelium [48]
Influence on vascular smooth muscle	miR‐145	KLF4, RhoA	Regulates SMC differentiation and function	Increased miR‐145 → modulation of vascular function → alters oxidative stress responses [49]

Abbreviations: ABCA1, ATP‐binding cassette transporter A1; Bcl2, B‐cell lymphoma 2; BNIP3, Bcl‐2/adenovirus E1B 19 kDa interacting protein 3; CAV1, caveolin‐1; CDK6, cyclin‐dependent kinase 6; EGFR, epidermal growth factor receptor; HDACs, histone deacetylases, enzymes involved in modifying histones and influencing gene expression; HO‐1, heme oxygenase‐1, an enzyme that helps in the breakdown of heme and has antioxidant properties; IRAK1, interleukin‐1 receptor‐associated kinase 1; KLF4, kruppel‐like factor 4; KRIT1, kristin 1; MDM2, mouse double minute 2 homolog; Nrf2, nuclear factor erythroid 2‐related factor 2 is a transcription factor that regulates antioxidant proteins; PGC1‐α, peroxisome proliferator‐activated receptor gamma coactivator 1‐alpha; PPARγ, peroxisome proliferator‐activated receptor gamma; PTEN, phosphatase and tensin homolog; RhoA, ras homolog family member A; ROS, reactive oxygen species, which are chemically reactive molecules that can cause oxidative damage; SIRT1, sirtuin 1; SOD2, superoxide dismutase 2; SPRED1, sprouty‐related protein 1; TRAF6, TNF receptor‐associated factor 6; VEGF, vascular endothelial growth factor.

#### 3.2.2 Modulation of Antioxidant Defense Mechanisms

Several miRNAs have been identified as direct regulators of genes involved in antioxidant defense. miR‐92a has been found to inhibit heme oxygenase‐1 (HO‐1), an essential enzyme responsible for degrading heme to produce biliverdin and bilirubin, both of which exhibit antioxidant properties [50]. By inhibiting miR‐92a, levels of HO‐1 increase, thereby enhancing the antioxidant capacity of endothelial cells and mitigating damage induced by oxidative stress. This demonstrates that miR‐92a serves as a negative regulator of antioxidant defenses, establishing a connection between its expression and oxidative stress levels [51].

#### 3.2.3 Regulation of ROS‐Producing Enzymes

Conversely, certain miRNAs can promote oxidative stress by targeting genes that regulate the production of ROS. miR‐21 is associated with increased ROS accumulation through its direct targeting of Krev/Rap1 interaction trapped‐1 (KRIT1) and superoxide dismutase 2 (SOD2), both crucial for maintaining redox homeostasis [52]. By downregulating these protective genes, miR‐21 contributes to elevated superoxide levels, particularly under high glucose conditions, thus illustrating its role in exacerbating oxidative stress [53].

#### 3.2.4 Interaction With Key Signaling Pathways

miRNAs also influence critical signaling pathways involved in oxidative stress responses. A prominent example is the Nrf2/Keap1 pathway; under normal conditions, Nrf2 is retained in the cytoplasm by Keap1 [54]. However, oxidative stress causes the dissociation of this complex, facilitating Nrf2's translocation to the nucleus, where it activates the expression of antioxidant genes [55]. Several miRNAs, such as miR‐144 and miR‐200a, have been reported to target Nrf2 directly or its upstream regulators, thereby modulating the antioxidant response. This interaction reveals the intricate regulatory networks where miRNAs can either enhance or inhibit cellular defenses against oxidative stress [56].

#### 3.2.5 Epigenetic Modulation

The expression of miRNAs themselves can also be influenced by oxidative stress through epigenetic mechanisms. For instance, ROS can induce modifications in histone acetylation that impact miRNA gene expression [12]. In this context, miR‐466h‐5p has been shown to be upregulated by ROS through the inhibition of histone deacetylases (HDACs), resulting in increased apoptosis via the targeting of anti‐apoptotic genes. This exemplifies how oxidative stress affects not only gene targets but also the expression of regulatory miRNAs [57].

#### 3.2.6 Feedback Mechanisms

Interestingly, a feedback loop exists between ROS levels and specific miRNAs. Elevated ROS can enhance the expression of specific miRNAs, such as miR‐200c, which subsequently influences cellular responses to oxidative stress [58]. This reciprocal relationship suggests that targeting these miRNA pathways could represent promising therapeutic strategies for managing cardiovascular conditions associated with oxidative stress [59].

### 3.3 Differential Expression of miRNAs in CVDs

miRNAs are integral to the regulation of oxidative stress pathways in CVDs by modulating the expression of genes that are critical for redox homeostasis and cellular responses to oxidative stress [33]. Numerous studies have elucidated specific miRNAs with altered expression profiles in various cardiovascular conditions, suggesting their potential utility as therapeutic targets and biomarkers (Table 2) [81].

**TABLE 2:** miRNAs regulating oxidative stress pathways in CVDs.

miRNA	Role in oxidative stress	Target genes	Associated conditions
miR‐1	Regulates cardiomyocyte growth and apoptosis; linked to oxidative stress responses	SOX6, Bcl2	Cardiac hypertrophy, myocardial infarction [60]
miR‐15	Involved in mitochondrial ROS production; regulates SIRT4	SIRT4	Ischemia/reperfusion injury [61]
miR‐21	Promotes oxidative stress; targets antioxidant genes	SOD2, PTEN	Hypertension, heart failure [53]
miR‐22	Regulates oxidative stress in cardiac tissues; upregulated in hypertrophy	SIRT1, PGC1‐α	Cardiac hypertrophy [62]
miR‐92a	Involved in angiogenesis; affects ROS levels in endothelial cells	HO‐1, VEGF	Atherosclerosis, ischemic heart disease [51, 63]
miR‐200 Family	Modulates endothelial dysfunction and redox balance	ZEB1, E‐cadherin	Diabetes‐related vascular complications [64, 65]
miR‐210	Enhances mitochondrial metabolism; reduces ROS sensitivity	HIF1A, PDK1	Hypoxia, ischemic conditions [66, 67]
miR‐499	Regulates cardiomyocyte viability and response to stress	SOX6	Heart failure, myocardial infarction [68]
miR‐130a	Stimulates angiogenesis; linked to endothelial function	Angiogenic factors	Atherosclerosis [69]
miR‐223	Modulates cholesterol homeostasis; influences inflammation	ABCA1, TNF‐α	Atherosclerosis [70]
miR‐27b	Promotes angiogenesis; linked to endothelial integrity and oxidative stress regulation	Various angiogenic factors	Atherosclerosis [71]
miR‐128	Identified as a “redoximiR”; modulates the expression of MAFG affecting antioxidant responses and cellular redox balance	MAFG	Oxidative stress‐related conditions [72, 73]
miR‐126	Implicated in endothelial protection; reduces oxidative stress	SPRED1, VEGF	Ischemic heart disease, diabetes [74]
miR‐146a	Involved in inflammation and oxidative stress responses; regulates NF‐κB pathway	IRAK1, TRAF6	Atherosclerosis, heart failure [75]
miR‐155	Modulates inflammation & oxidative stress; involved in macrophage activation	SOCS1, TNF‐α	Atherosclerosis, inflammatory heart disease [76]
miR‐29	Targets extracellular matrix components; involved in fibrosis and oxidative stress	COL1A1, PTEN	Heart failure, fibrosis [77]
miR‐30	Regulates autophagy and protects against oxidative stress	BNIP3, Beclin‐1	Cardiomyopathy, ischemic heart disease [78]
miR‐34a	Influences oxidative state; associated with cell cycle and apoptosis regulation	CDK6, Bcl2	Aging, heart failure [79]
miR‐122	Affects lipid metabolism and oxidative stress response	ACACB, SIRT1	Metabolic syndrome, atherosclerosis [80]

Abbreviations: ABCA1, ATP‐binding cassette subfamily a member 1; ACACB, acetyl‐CoA carboxylase beta; Bcl2, B‐cell lymphoma 2; Bcl2, B‐cell lymphoma 2; beclin‐1, Bcl‐2 interacting protein 1; BNIP3, Bcl‐2/adenovirus E1B 19 kDa interacting protein 3; CDK6, cyclin‐dependent kinase 6; COL1A1, collagen type I alpha 1; E‐cadherin, epithelial cadherin; HIF1A, hypoxia‐inducible factor 1 alpha; HO‐1, heme oxygenase 1; IRAK1, interleukin‐1 receptor‐associated kinase 1; MAFG, MAF BZIP transcription factor G; PDK1, pyruvate dehydrogenase kinase 1; PGC1‐α, peroxisome proliferator; PTEN, phosphatase and tensin homolog; PTEN, phosphatase and tensin homolog; SIRT1, sirtuin 1; SIRT1, sirtuin 1; SIRT4, sirtuin 4; SOCS1, suppressor of cytokine signaling 1; SOD2, superoxide dismutase 2; SOX6, SRY‐box transcription factor 6; SPRED1, sprouty‐related protein 1; TNF‐α, tumor necrosis factor alpha; TNF‐α, tumor necrosis factor‐alpha; TRAF6, TNF receptor‐associated factor 6; VEGF, vascular endothelial growth factor; VEGF, vascular endothelial growth factor; ZEB1, zinc finger E.

miR‐210 is markedly upregulated under hypoxic conditions, demonstrating protective effects on endothelial cells against apoptosis induced by oxidative stress [66]. In human umbilical vein endothelial cells (HUVECs) exposed to hydrogen peroxide (H_2_O_2_), miR‐210 overexpression led to a decrease in ROS levels and downregulation of pro‐apoptotic factors such as caspases, ultimately promoting cell survival [67].

miR‐21 is associated with elevated ROS levels; miR‐21 is implicated in endothelial dysfunction. It targets key genes involved in maintaining ROS homeostasis, such as KRIT1 and SOD2, resulting in increased oxidative stress when overexpressed [53]. Research indicates that miR‐21 facilitates the accumulation of superoxide in cells, particularly in the context of high glucose concentrations, which is particularly relevant in diabetic vascular complications [53].

Specific members of the miR‐200 family, particularly miR‐200c, exhibit significant upregulation in response to oxidative stress [64]. These miRNAs play critical roles in endothelial cell reactions to ROS by regulating inflammatory and apoptotic pathways. The induction of miR‐200c has been associated with enhanced endothelial function and reduced oxidative damage, indicating its protective role in CVDs [65].

miR‐128 also known as a “redoximiR,” directly targets MAF BZIP Transcription Factor G (MAFG), a transcription factor involved in the regulation of antioxidant genes [72]. By modulating MAFG expression, miR‐128 influences the activation of antioxidant responses mediated by antioxidant‐responsive elements (ARE), thereby affecting the cellular redox balance [73].

miR‐92a, as part of the miR‐17‐92 cluster, negatively regulates heme oxygenase 1 (HO‐1), an enzyme critical for the degradation of heme and the production of biliverdin and bilirubin, both of which act as potent antioxidants [51]. The inhibition of miR‐92a results in enhanced expression of HO‐1, leading to a reduction in oxidative stress and the preservation of endothelial function [63].

The differential expression of these miRNAs underscores their complex roles in modulating oxidative stress pathways within the cardiovascular system. Their regulatory influence on target genes associated with ROS production and detoxification highlights the potential for utilizing miRNAs as therapeutic agents or biomarkers for CVD related to oxidative stress [33]. A deeper understanding of these interactions will illuminate the intricate network between miRNAs and oxidative stress, paving the way for innovative strategies for the effective management of CVDs.

### 3.4 TheranoMiRNAs

The concept of “theranoMiRNAs” has emerged as a significant innovation in the conception of miRNA dysregulation in CVDs [82]. This term refers to the dual functionality of miRNAs, which can serve as both diagnostic biomarkers and therapeutic agents. Their dual role as biomarkers and therapeutic agents enables a more targeted and individualized approach to disease detection and treatment [83, 84].

In cardiovascular medicine, the application of theranoMiRNAs is particularly promising, given their involvement in critical processes such as endothelial function, myocardial remodeling, and atherosclerosis progression [85]. Researchers and clinicians can use their theragnostic properties to create innovative approaches for early CVD detection, risk stratification, and personalized treatment [86]. Additionally, advancements in RNA delivery technologies, such as lipid nanoparticles, viral vectors, and exosome‐based systems, are further enhancing the feasibility of miRNA‐based interventions, positioning theranoMiRNAs as a transformative tool in next‐generation cardiovascular therapeutics [87].

#### 3.4.1 TheranoMiRNAs in Cardiovascular Disease

Among the most well‐characterized theranoMiRNAs in cardiovascular disease (CVD), miR‐126 stands out due to its critical involvement in vascular integrity, angiogenesis, and endothelial repair. Circulating levels of miR‐126 have been shown to decrease in patients with atherosclerosis and coronary artery disease, positioning it as a valuable biomarker for early diagnosis and risk stratification in vascular disorders [88]. Therapeutically, miR‐126 mimics have been explored to enhance endothelial regeneration and promote angiogenesis, offering a potential treatment strategy for ischemic cardiovascular diseases by restoring vascular integrity and function [89].

miR‐21 represents another prominent theranoMiRNA with dual roles in CVD. This miRNA is significantly upregulated in conditions such as cardiac fibrosis and heart failure, where it serves as a key biomarker of disease progression and myocardial injury [90]. On the therapeutic front, miR‐21 inhibitors (antagomiRs) have shown promise in mitigating fibrosis, improving myocardial stiffness, and enhancing cardiac function, presenting it as an important target for therapeutic intervention in fibrotic heart disease and post‐infarction remodeling [91].

miR‐34a has emerged as a promising theranomiRNA due to its significant role in cardiomyocyte apoptosis and age‐related cardiac dysfunction. Elevated circulating levels of miR‐34a are strongly associated with worsening heart failure and adverse cardiac remodeling, making it an effective diagnostic marker for disease severity [92]. Additionally, miR‐34a inhibitors have demonstrated the ability to prevent cardiac cell death and promote cardiac regeneration, further establishing miR‐34a's theragnostic potential for improving outcomes in heart failure and chronic cardiac conditions [93].

Also, among the extensively studied theranoMiRNAs, miR‐133a and miR‐208a are more known as key biomarkers for acute myocardial infarction (AMI), released into the circulation during myocardial injury and correlating with necrosis. Their expression patterns enable rapid AMI diagnosis, potentially surpassing traditional biomarkers like troponins [94, 95]. miR‐126, a regulator of endothelial function, plays a role in vascular integrity and repair, with altered levels linked to atherosclerosis and ischemic injury, making it a promising candidate for risk assessment and therapeutic intervention [96].

On the therapeutic side, miR‐21, upregulated in cardiac fibrosis, contributes to ECM remodeling and fibrosis. AntagomiRs targeting miR‐21 have shown potential in reducing fibrosis and improving cardiac function in heart failure and post‐infarction remodeling [97]. Similarly, miR‐92a, implicated in endothelial dysfunction and atherogenesis, has been identified as a key factor in vascular health [98].

### 3.5 Clinical Implications of miRNAs in CVDs

miRNAs have emerged as fundamental regulators in the pathophysiology of CVDs, presenting promising opportunities for diagnosis, prognosis, and therapeutic intervention [81]. Their capacity to modulate gene expression at the post‐transcriptional level positions them as critical contributors to various cardiovascular conditions, including heart failure, myocardial infarction, and arrhythmias [99].

#### 3.5.1 Potential as Biomarkers

The stability and specific tissue expression patterns of miRNAs make them valuable biomarkers for diagnosing and prognosticating CVDs [100].

**FIGURE 1:**
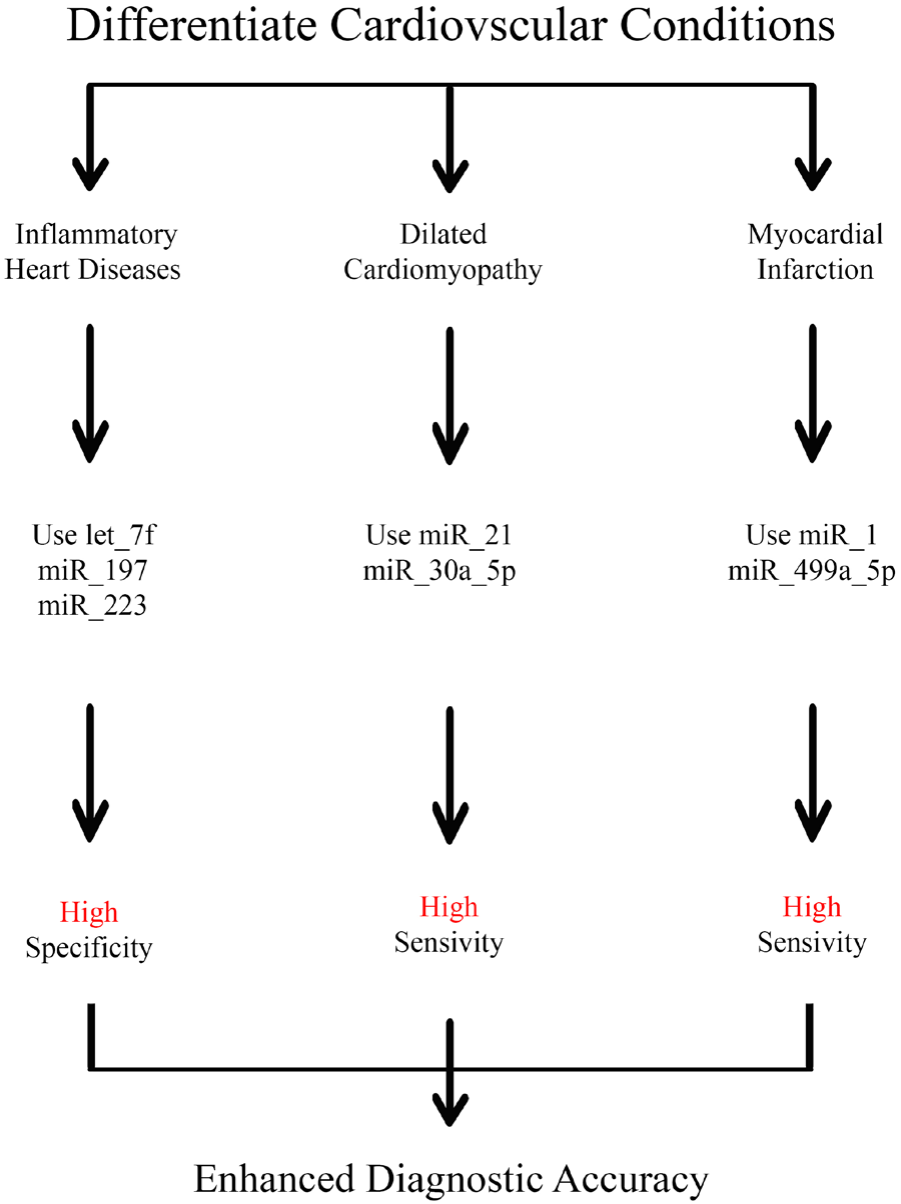
MiRNA biomarkers in cardiovascular disease. miRNAs let‐7f, miR‐197, and miR‐223 can distinguish between conditions such as myocardial infarction (MI) and inflammatory heart diseases, with over 93% specificity. Higher levels of miR‐21 and miR‐30a‐5p are linked to dilated cardiomyopathy. miR‐1 and miR‐499a‐5p show increased expression shortly after MI, offering greater sensitivity and specificity than traditional biomarkers.

Recent studies have demonstrated that distinct miRNA expression profiles can effectively differentiate among various cardiovascular conditions, such as myocardial infarction (MI), heart failure, and inflammatory heart diseases. One study identified a panel of circulating miRNAs, including let‐7f, miR‐197, and miR‐223, successfully distinguishing patients with inflammatory heart diseases from healthy individuals, achieving over 93% specificity [101]. Similarly, elevated levels of miR‐21 and miR‐30a‐5p were linked to dilated cardiomyopathy, enabling accurate patient classification based on their miRNA expression profiles [102]. Additional studies have accentuated the diagnostic potential of miRNAs like miR‐1 and miR‐499a‐5p, which exhibited increased expression in MI patients within hours of symptom onset, surpassing traditional biomarkers such as cardiac troponins in terms of sensitivity and specificity (Figure 1) [103].

The ability of miRNAs to reflect pathological changes through their expression levels in biofluids like serum and plasma positions them as essential tools for the early diagnosis and monitoring of CVD progression [81]. Furthermore, combining miRNA profiles with existing biomarkers could enhance diagnostic accuracy and provide insights into patient prognosis, ultimately improving clinical decision‐making [104].

#### 3.5.2 Therapeutic Targets

**FIGURE 2:**
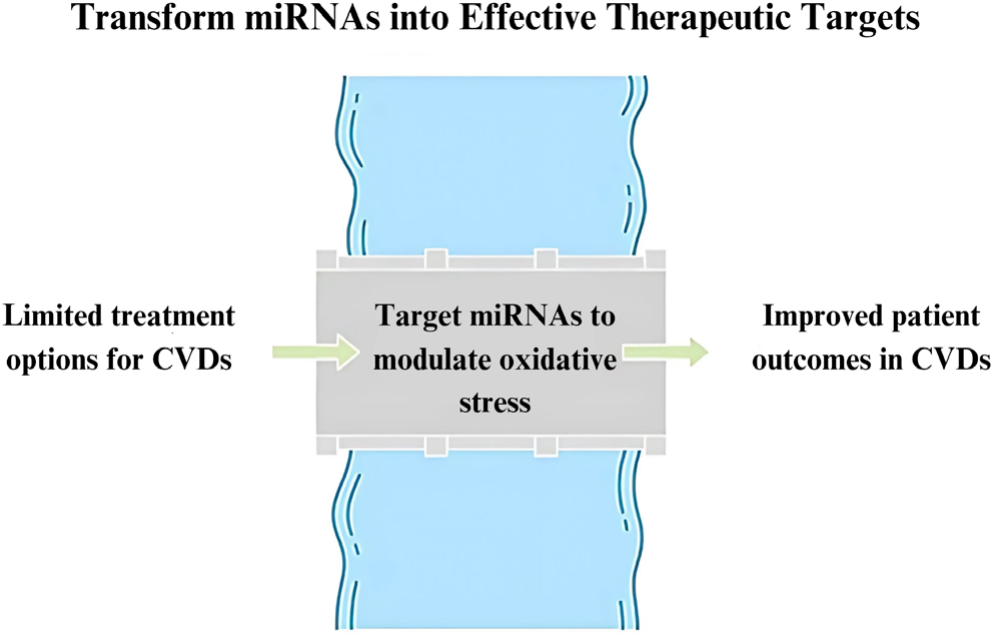
Therapeutic targets in CVDs. miRNAs can be targeted to reduce oxidative stress in CVDs, enhancing antioxidant defenses and endothelial function. Additionally, synthetic miRNA mimics or inhibitors may restore redox balance and improve resilience in heart cells.

Beyond their diagnostic applications, miRNAs represent novel therapeutic targets for addressing oxidative stress in CVDs. Given their roles in regulating gene expression associated with oxidative stress pathways, the modulation of specific miRNAs could yield innovative therapeutic strategies [33]. Strategies targeting the upregulated miR‐21 have been proposed to counteract its pro‐oxidative effects in endothelial cells. Inhibiting miR‐21 may restore the expression of antioxidant genes such as SOD2, thereby reducing ROS levels and enhancing endothelial function [105]. Similarly, downregulating miR‐92a could increase levels of heme oxygenase 1 (HO‐1), which would bolster antioxidant defenses against oxidative stress [106]. Moreover, the clinical application of synthetic miRNA mimics or inhibitors shows promise for reestablishing normal redox balance within cardiovascular tissues [107]. Recent studies have demonstrated that delivering miR‐210 mimics could improve cellular resilience against oxidative stress by enhancing mitochondrial function and reducing apoptotic pathways in cardiomyocytes subjected to hypoxic conditions (Figure 2) [108]. Despite the significant therapeutic potential of utilizing miRNAs as targets, challenges persist regarding delivery methods and the risk of off‐target effects. Future research endeavors should concentrate on developing effective delivery systems to ensure the precise action of miRNA‐based therapies while minimizing adverse impacts on non‐target tissues [109].

## 4 Importance of In Silico Studies in miRNA Research

In silico studies have become an invaluable tool in the field of molecular biology, particularly in understanding the complex interactions between miRNAs and their target genes. These computational approaches enable researchers to predict miRNA‐target interactions, elucidate regulatory networks, and explore the functional implications of miRNA dysregulation in various diseases, including cardiovascular diseases (CVDs) [110, 111].

### 4.1 Predictive Modeling of miRNA‐Target Interactions

Computational tools such as TargetScan, miRDB, and DIANA‐microT provide platforms for predicting potential targets of specific miRNAs based on sequence complementarity and conservation across species. These predictions are crucial for identifying the downstream effects of miRNAs on gene expression and understanding their roles in oxidative stress pathways [112, 113]. using these tools, researchers have identified key genes involved in ROS production and detoxification that are regulated by specific miRNAs, thereby providing insights into their mechanistic roles [113].

### 4.2 Validation of Experimental Findings

In silico analyses can complement experimental data by validating findings from laboratory studies. By cross‐referencing predicted targets with experimentally validated interactions from databases such as TarBase and miRTarBase, researchers can strengthen the credibility of their results. This validation process is essential for ensuring that the reported effects of miRNAs on oxidative stress are based on robust evidence [114].

### 4.3 Insights Into Pathophysiological Mechanisms

In silico studies also enable a deeper understanding of the pathophysiological mechanisms underlying CVDs related to oxidative stress. By integrating data from various omics technologies (e.g., genomics, transcriptomics), researchers can construct comprehensive models that depict how miRNAs influence cellular responses to oxidative stress at multiple levels. This approach facilitates the identification of novel biomarkers for early diagnosis and potential therapeutic targets [23].

### 4.4 Computational Drug Design

Furthermore, in silico methods play a pivotal role in drug discovery and development processes aimed at targeting miRNA pathways. Computational modeling can help design miRNA mimics or inhibitors that modulate specific pathways implicated in oxidative stress, paving the way for innovative therapeutic strategies [115].

The integration of in silico studies into miRNA research significantly enhances our understanding of their regulatory roles in oxidative stress within CVDs [116]. As computational tools continue to evolve, they will undoubtedly provide valuable insights that complement experimental findings and contribute to the advancement of precision medicine approaches targeting oxidative damage [116].

## 5 Conclusions

The interplay between miRNAs and oxidative stress represents a complex and dynamic relationship that is critical for understanding the pathophysiology of CVDs. This review has highlighted several key findings regarding the regulatory roles of miRNAs in oxidative stress pathways, emphasizing their potential as both diagnostic biomarkers and therapeutic targets. Specific miRNAs, such as miR‐21 and miR‐210, exhibit altered expression profiles in response to oxidative stress, making them promising candidates for biomarkers that could aid in early diagnosis and risk stratification. Elevated levels of miR‐21 have been associated with adverse cardiac remodeling, while miR‐210 levels correlate with improved endothelial function under hypoxic conditions. On the therapeutic front, strategies aimed at modulating miRNA expression—such as the use of miRNA mimics or inhibitors—hold promise for restoring normal cellular functions disrupted by oxidative stress, potentially leading to innovative treatments targeting specific molecular pathways involved in oxidative damage. However, several limitations must be acknowledged, including interindividual variability in miRNA expression influenced by genetic, environmental, and lifestyle factors, which complicates the establishment of standardized diagnostic criteria. Additionally, translating findings from preclinical models into clinical applications remains a significant difficulty due to challenges in developing effective delivery systems for miRNA‐based therapies that ensure stability and specificity in vivo. Technological advancements are improving the conception and application of miRNA research in CVDs. In silico approaches, such as those provided by TargetScan and DIANA‐microT, have become invaluable for predicting miRNA‐target interactions and validating experimental findings, while novel methodologies like CRISPR/Cas9 gene editing offer exciting possibilities for directly manipulating miRNA expression or function within specific cell types or tissues. These technologies could significantly advance our ability to study the functional roles of miRNAs in oxidative stress pathways and their implications for cardiovascular health. In summary, this review underscores the critical roles that miRNAs play in regulating oxidative stress within CVDs. While challenges remain regarding their clinical application as biomarkers and therapeutics, Further research into their mechanisms and interactions will be essential for unlocking their full potential. Future studies should focus on addressing current limitations while exploring innovative technologies to enhance our knowledge of miRNA biology in CVDs.

## Author Contributions

Sakhavat Abolhasani and Yasin Ahmadi primarily developed the study. Yavar Rostami contributed by focusing on editing and providing resource support, while Khalil Maleki Chollou also assisted with editing and resource support. Davood Fattahi was responsible for the final revision of the manuscript. All authors collaborated to write the manuscript, with Sakhavat Abolhasani and Davood Fattahi overseeing the writing process.

## Ethics Statement

The authors have nothing to report.

## Consent

The authors have nothing to report.

## Conflicts of Interest

The authors declare no conflicts of interest.

## Supporting information


Appendix S1.

## Data Availability

The datasets gathered and examined in this study can be obtained from the corresponding author upon a reasonable request. Additionally, the names of the repositories and their reference numbers are accessible in online repositories.
