# Supplementary material for: microRNA‐Mediated Regulation of Oxidative Stress in Cardiovascular Diseases
Source: J Clin Lab Anal. 2025 Apr 4;39(9):e70017. doi: 10.1002/jcla.70017 (PMC12078765; doi:10.1002/jcla.70017)
Supplement: Supplementary file 1 — Appendix S1. [file JCLA-39-e70017-s001.docx]

**The predicted target genes of various microRNAs, including miR-210, miR-21, the miR-200 family, miR-128, and miR-92a, play crucial roles in the regulation of oxidative stress and are implicated in the pathogenesis or prevention of cardiovascular diseases.**

**The miRNA target prediction data were extracted from miRDB (**[**http://mirdb.org**](http://mirdb.org)**), a reliable online database that provides miRNA-target interaction predictions based on machine learning algorithms.**

**Target Rank** indicates the relative position of a gene's predicted interaction with a miRNA, with lower ranks signifying stronger, more reliable interactions.

**Target Score** quantifies the strength or confidence of this interaction, with higher scores reflecting stronger predictions.

(a lower **Target Rank** and a higher **Target Score** together suggest a more prominent and confident miRNA-gene interaction).

**1) Likely Targeted Genes (out of 401 predicted targets)** **by hsa-miR-210-5p in miRDB Involved in Oxidative Stress Regulation and Cardiovascular Diseases**

| **Target Rank** | **Target Score** | **Gene Description** |
| --- | --- | --- |
| 20 | 87 | **AKT serine/threonine kinase 3** (Regulates cell survival and oxidative stress responses; dysfunction promotes endothelial dysfunction and CVD) |
| 24 | 86 | **LDL receptor related protein 4** (Modulates lipid metabolism and oxidative stress; altered function contributes to atherosclerosis) |
| 26 | 85 | **Dehydrogenase/reductase X-linked** (Involved in redox homeostasis; dysfunction increases oxidative stress and promotes vascular damage) |
| 29 | 85 | **Fas associated factor family member 2** (Regulates apoptosis and inflammation; oxidative stress enhances its role in endothelial injury) |
| 41 | 83 | **Chromosome 20 open reading frame 202** (Impacts oxidative stress pathways; dysregulation may influence vascular inflammation and remodeling) |
| 49 | 81 | **Upstream transcription factor 1** (Regulates antioxidant defense and lipid metabolism; altered function contributes to CVD progression) |
| 53 | 80 | **Protein tyrosine phosphatase, receptor type D** (Modulates endothelial function and oxidative stress response; its dysregulation promotes vascular disease) |
| 79 | 76 | **Serum response factor** (Regulates vascular smooth muscle cell function; oxidative stress affects its role in vascular remodeling) |
| 115 | 71 | **Phospholipase A2 group XV** (Involved in lipid oxidation and inflammation; oxidative stress enhances its contribution to endothelial dysfunction) |
| 121 | 71 | **NKD1, WNT signaling pathway inhibitor** (Modulates oxidative stress and inflammatory responses; dysregulation impacts vascular homeostasis) |

**2) Likely Targeted Genes (out of 83 predicted targets)** **by hsa-miR-210-3p in miRDB Involved in Oxidative Stress Regulation and Cardiovascular Diseases**

| **Target Rank** | **Target Score** | **Gene Description** |
| --- | --- | --- |
| 1 | 93 | **IGF2** (Implicated in cellular proliferation and differentiation, oxidative stress regulation, and CVD via survival/apoptosis pathways) |
| 5 | 88 | **AIFM3** (Involved in mitochondrial regulation and apoptosis, linked to oxidative stress and heart function in CVD) |
| 7 | 87 | **FGFRL1** (Affects vascular smooth muscle cells and vascular remodeling, regulated by oxidative stress in CVD) |
| 9 | 83 | **BDNF** (Regulates neuronal and cardiac function, involved in oxidative stress response mechanisms and linked to CVD) |
| 15 | 76 | **SDF2** (Involved in inflammation and tissue injury, modulating oxidative stress and inflammation in CVD) |
| 16 | 76 | **ACVR1B** (Plays a role in fibrosis, vascular remodeling, inflammation, and apoptosis, influenced by oxidative stress in CVD) |
| 18 | 75 | **THSD7A** (Involved in cell adhesion, extracellular matrix remodeling, and vascular inflammation, impacted by oxidative stress in CVD) |

**3) Likely Targeted Genes (out of 595 predicted targets)** **by hsa-miR-21-3p in miRDB Involved in Oxidative Stress Regulation and Cardiovascular Diseases**

| **Target Rank** | **Target Score** | **Gene Description** |
| --- | --- | --- |
| 301 | 68 | **RNA binding motif protein 33** (Regulates gene expression in response to oxidative stress, influencing cardiovascular pathophysiology) |
| 302 | 68 | **Fibroblast growth factor receptor substrate 2** (Involved in signaling pathways that modulate inflammation and oxidative stress, contributing to vascular remodeling in CVD) |
| 303 | 68 | **Neurocalcin delta** (Modulates intracellular calcium levels, which are critical in oxidative stress responses, and contributes to vascular dysfunction in CVD) |
| 304 | 68 | **Zinc finger protein 101** (Regulates cellular response to oxidative stress, influencing endothelial function and the progression of atherosclerosis) |
| 305 | 68 | **Semaphorin 3E** (Modulates vascular remodeling and angiogenesis; oxidative stress impairs its function, contributing to CVD) |
| 306 | 68 | **Parkin coregulated like** (Regulates mitochondrial function and mitophagy, and oxidative stress leads to mitochondrial dysfunction, affecting CVD development) |
| 307 | 68 | **Zinc finger protein 250** (Influences DNA repair and cellular stress responses; its dysfunction can lead to increased oxidative stress, promoting CVD) |
| 308 | 68 | **Nuclear FMR1 interacting protein 2** (Modulates responses to oxidative stress; altered function can contribute to vascular disease progression) |
| 309 | 68 | **ST6 N-acetylgalactosaminide alpha-2,6-sialyltransferase 5** (Involved in glycosylation; oxidative stress affects its activity, influencing endothelial cell function and CVD progression) |
| 310 | 67 | **Triple QxxK/R motif containing** (Involved in cellular stress response pathways; dysfunction under oxidative stress promotes vascular damage and CVD) |
| 311 | 67 | **Aryl hydrocarbon receptor nuclear translocator like** (Modulates oxidative stress and inflammation, influencing endothelial function and CVD development) |
| 312 | 67 | **Sphingosine-1-phosphate receptor 3** (Involved in regulating vascular tone and inflammation; oxidative stress disrupts its activity, contributing to CVD) |
| 313 | 67 | **Syndecan 2** (Modulates extracellular matrix remodeling; oxidative stress impairs its function, contributing to vascular stiffness in CVD) |
| 314 | 67 | **NIPA magnesium transporter 1** (Regulates magnesium homeostasis; oxidative stress affects magnesium levels, leading to endothelial dysfunction and hypertension in CVD) |
| 315 | 67 | **Transferrin receptor** (Regulates iron uptake; oxidative stress exacerbates iron overload, contributing to endothelial damage and CVD) |
| 316 | 67 | **BAALC, MAP3K1 and KLF4 binding** (Involved in cell differentiation and stress responses; altered function can exacerbate oxidative damage and accelerate CVD) |
| 317 | 67 | **Lipopolysaccharide induced TNF factor** (Modulates inflammation and oxidative stress; dysregulation contributes to the inflammatory response in CVD) |
| 318 | 67 | **Beta-1,4-galactosyltransferase 6** (Modulates cellular stress responses; alterations under oxidative stress influence vascular remodeling and CVD progression) |
| 319 | 67 | **Plexin domain containing 2** (Involved in cell signaling and vascular development; oxidative stress alters its function, contributing to vascular dysfunction in CVD) |
| 320 | 67 | **Cbl proto-oncogene B** (Regulates cell survival and apoptosis; oxidative stress disrupts its function, promoting vascular injury and CVD) |
| 321 | 67 | **Mitogen-activated protein kinase kinase kinase 7** (Involved in oxidative stress response pathways; dysregulation contributes to inflammation and endothelial dysfunction in CVD) |
| 322 | 67 | **Grainyhead like transcription factor 1** (Regulates cellular responses to oxidative stress; altered function can contribute to vascular injury and CVD progression) |
| 323 | 66 | **Angiogenic factor with G-patch and FHA domains 1** (Modulates angiogenesis; oxidative stress impairs its function, disrupting vascular health and promoting CVD) |
| 324 | 66 | **Iodothyronine deiodinase 2** (Modulates thyroid hormone metabolism; oxidative stress alters its activity, influencing cardiovascular function) |
| 325 | 66 | **Arginine and serine rich coiled-coil 2** (Involved in cellular response to stress; dysregulation under oxidative stress contributes to endothelial dysfunction in CVD) |
| 326 | 66 | **Olfactomedin 3** (Regulates cellular stress responses; its dysfunction under oxidative stress contributes to vascular damage and CVD) |
| 327 | 66 | **Transmembrane protein 47** (Involved in cellular stress and oxidative responses; its dysfunction can lead to vascular injury and contribute to CVD) |
| 328 | 66 | **Vacuolar protein sorting 13 homolog C** (Modulates cellular responses to stress; altered function under oxidative stress influences vascular function and contributes to CVD) |
| 329 | 66 | **Pre-mRNA processing factor 38B** (Regulates RNA processing; oxidative stress can impair its function, influencing gene expression and contributing to CVD) |
| 330 | 66 | **Leucine rich repeat containing 17** (Regulates stress response pathways; dysregulation leads to increased oxidative stress and contributes to CVD) |
| 331 | 66 | **Stannin** (Involved in stress response regulation; dysfunction under oxidative stress can promote vascular damage and CVD progression) |
| 332 | 66 | **SCY1 like pseudokinase 2** (Modulates cellular stress responses; dysregulation influences oxidative stress pathways and CVD development) |
| 333 | 66 | **Protein O-fucosyltransferase 2** (Involved in stress signaling; oxidative stress disrupts its function, contributing to vascular injury and CVD) |
| 334 | 66 | **TEA domain transcription factor 1** (Modulates gene expression in response to stress; oxidative stress can alter its activity, contributing to endothelial dysfunction and CVD) |

**4) Likely Targeted Genes (out of 469 predicted targets)** **by hsa-miR-21-5p in miRDB Involved in Oxidative Stress Regulation and Cardiovascular Diseases**

| **Target Rank** | **Target Score** | **Gene Description** |
| --- | --- | --- |
| 3 | 99 | **Fas ligand** (Regulates apoptosis, contributing to endothelial dysfunction and atherosclerosis under oxidative stress) |
| 9 | 97 | **Interleukin 12A** (Modulates inflammatory and oxidative stress responses, influencing cardiovascular pathology) |
| 27 | 94 | **Signal transducer and activator of transcription 3** (Involved in oxidative stress-induced inflammation and cardiac remodeling) |
| 36 | 93 | **NFKB inhibitor interacting Ras-like 1** (Regulates NF-κB signaling, modulating oxidative stress and inflammation in CVD) |
| 45 | 92 | **Mitogen-activated protein kinase kinase kinase 1** (Involved in oxidative stress signaling, contributing to cardiac hypertrophy and vascular remodeling) |
| 54 | 91 | **TIMP metallopeptidase inhibitor 3** (Protects against oxidative stress-induced extracellular matrix degradation and cardiac fibrosis) |
| 57 | 91 | **Jagged 1** (Regulates vascular development; oxidative stress impairs its function, promoting endothelial dysfunction in CVD) |
| 112 | 84 | **Lipoprotein(a)** (Pro-atherogenic factor; oxidative stress enhances its role in vascular inflammation and plaque formation) |

**5) Likely Targeted Genes (out of 1088 predicted targets)** **by hsa-miR-200a-3p in miRDB Involved in Oxidative Stress Regulation and Cardiovascular Diseas**

| **Target Rank** | **Target Score** | **Gene Description** |
| --- | --- | --- |
| 596 | 71 | **Oxidative stress induced growth inhibitor family member 2**: (Involved in the cellular response to oxidative stress, regulating growth inhibition. Plays a role in endothelial dysfunction and vascular remodeling in CVD.) |
| 467 | 77 | **Mitogen-activated protein kinase 6 (MAPK6)**: (Regulates stress responses, including oxidative stress. Involved in inflammation and vascular smooth muscle cell proliferation in CVD.) |
| 495 | 76 | **Mitogen-activated protein kinase kinase kinase 7 (MAP3K7)**: (Key regulator of oxidative stress signaling pathways. Implicated in inflammatory responses and endothelial cell dysfunction in CVD.) |
| 487 | 76 | **Transforming growth factor beta receptor 1 (TGFBR1)**: (Regulates vascular remodeling and fibrosis. Modulates oxidative stress, contributing to the pathogenesis of atherosclerosis and CVD.) |
| 475 | 76 | **Protein phosphatase 1 regulatory subunit 15B (PPP1R15B)**: (Involved in cellular stress responses, including oxidative stress. Regulates endothelial cell apoptosis and inflammation in CVD.) |
| 521 | 74 | **Leucine rich repeat containing 8 VRAC subunit B (LRRC8B)**: (Plays a role in cellular responses to osmotic stress and oxidative stress. Involved in vascular smooth muscle cell function in CVD.) |
| 522 | 74 | **Activating transcription factor 6 (ATF6)**: (Regulates the unfolded protein response and oxidative stress responses. Involved in endothelial cell survival and inflammation in CVD.) |
| 506 | 75 | **Zinc finger protein 281 (ZNF281)**: (Regulates transcription factors involved in cellular stress responses. Plays a role in the regulation of oxidative stress in vascular cells in CVD.) |
| 534 | 74 | **Pyridine nucleotide-disulphide oxidoreductase domain 1 (NDUFS1)**: (Involved in mitochondrial function and oxidative stress regulation. Contributes to oxidative damage and endothelial dysfunction in CVD.) |
| 23 | 98 | **Mitogen-activated protein kinase kinase 4 (MAP2K4)**: (Involved in the MAPK signaling pathway, which regulates oxidative stress and inflammation, contributing to CVD development.) |
| 27 | 98 | **Cyclin dependent kinase 8 (CDK8)**: (Controls cell cycle and transcription factors linked to oxidative stress, influencing cardiovascular health.) |
| 34 | 97 | **Protein kinase C epsilon (PRKCE)**: (Plays a role in cardiac function and response to oxidative stress, impacting CVD outcomes.) |
| 76 | 95 | **Transforming growth factor beta 2 (TGFB2)**: (Modulates oxidative stress and fibrosis in cardiovascular tissues, contributing to disease progression.) |
| 910 | 57 | **NADPH oxidase 1**: (Involved in the production of reactive oxygen species (ROS) and plays a key role in oxidative stress, which contributes to vascular injury and inflammation in CVD.) |
| 920 | 57 | **NADPH oxidase 5**: (Similar to NADPH oxidase 1, it generates ROS, promoting oxidative damage and endothelial dysfunction, important in the pathogenesis of CVD.) |
| 813 | 61 | **Transmembrane protein 255A**: (Though its exact role is unclear, it is believed to influence oxidative stress pathways and has been associated with atherosclerosis and vascular diseases.) |
| 910 | 57 | **NADPH oxidase 1**: (Involved in ROS production, leading to vascular inflammation and endothelial dysfunction in cardiovascular diseases.) |
| 907 | 57 | **Family with sequence similarity 84 member A**: (Contributes to oxidative stress by regulating redox-sensitive proteins, influencing cardiovascular inflammation.) |

**6) Likely Targeted Genes (out of 438 predicted targets)** **by hsa-miR-200a-5p in miRDB Involved in Oxidative Stress Regulation and Cardiovascular Diseas**e

| **Target Rank** | **Target Score** | **Gene Description and Role in Oxidative Stress and CVD** |
| --- | --- | --- |
| 30 | 92 | **Thioredoxin reductase 3**: This enzyme plays a crucial role in regulating cellular redox balance, reducing oxidative stress by maintaining thiol-disulfide equilibrium. It protects against endothelial dysfunction and atherosclerosis in cardiovascular diseases (CVD). |
| 36 | 91 | **Peroxisome proliferator-activated receptor gamma, coactivator-related 1**: This gene regulates oxidative stress responses by modulating cellular metabolism and inflammatory pathways. It is linked to insulin resistance and plays a role in increasing CVD risk through inflammation and oxidative damage. |
| 92 | 82 | **Electron transfer flavoprotein dehydrogenase**: This enzyme is involved in mitochondrial energy production and maintaining redox homeostasis. Its dysfunction can lead to an accumulation of oxidative stress, contributing to heart disease progression and vascular damage. |
| 92 | 82 | **Glutaminase**: Glutaminase regulates glutamine metabolism, influencing the cellular response to oxidative stress. Its role in CVD includes the modulation of oxidative stress in endothelial cells, affecting vascular health and contributing to disease mechanisms. |
| 91 | 82 | **Thioredoxin domain containing 17**: This gene is crucial for maintaining cellular redox balance by controlling thiol-disulfide exchange, helping to reduce oxidative damage in vascular cells and preventing the development of cardiovascular diseases through its antioxidant activity. |

**7) Likely Targeted Genes (out of 1294 predicted targets)** **by hsa-miR-200b-3p in miRDB Involved in Oxidative Stress Regulation and Cardiovascular Diseas**e

| **Target Rank** | **Target Score** | **Gene Description** |
| --- | --- | --- |
| 1 | 100 | **Vasohibin 2** (Involved in the regulation of angiogenesis and vascular remodeling, where oxidative stress plays a crucial role in endothelial dysfunction in CVD). |
| 5 | 100 | **Zinc finger E-box binding homeobox 1 (ZEB1)** (Regulates transcription factors linked to oxidative stress, vascular inflammation, and endothelial cell dysfunction in CVD). |
| 6 | 100 | **Nuclear receptor subfamily 5 group A member 2 (NR5A2)** (Modulates oxidative stress response pathways and lipid metabolism, contributing to atherosclerosis and CVD). |
| 9 | 99 | **WAS/WASL interacting protein family member 1** (Regulates actin dynamics and oxidative stress response, implicated in endothelial cell function in cardiovascular diseases). |
| 11 | 99 | **Chromosome 11 open reading frame 95** (Regulates oxidative stress and inflammation, key processes in atherosclerosis and CVD). |
| 13 | 99 | **LHFPL tetraspan subfamily member 6** (Modulates oxidative stress, playing a role in vascular smooth muscle cell function in CVD). |
| 17 | 99 | **Protein tyrosine phosphatase, non-receptor type 21 (PTPN21)** (Involved in inflammatory signaling pathways and regulation of oxidative stress in cardiovascular diseases). |
| 23 | 99 | **Cofilin 2** (Regulates actin filaments and cell signaling, impacting endothelial cell response to oxidative stress in cardiovascular diseases). |
| 25 | 98 | **Rho GTPase activating protein 6 (ARHGAP6)** (Modulates oxidative stress pathways and regulates vascular smooth muscle cell contraction in CVD). |
| 28 | 98 | **BRCA1 associated protein 1 (BAP1)** (Involved in DNA repair and oxidative stress regulation, impacting vascular function in CVD). |
| 36 | 98 | **WAS protein family member 3 (WASF3)** (Regulates actin polymerization and redox signaling, influencing vascular function and CVD progression). |
| 38 | 97 | **Elongation factor for RNA polymerase II 2 (EP300)** (Modulates transcriptional activity in response to oxidative stress, influencing cardiovascular disease development). |
| 43 | 97 | **Casein kinase 1 gamma 3 (CSNK1G3)** (Regulates oxidative stress and inflammatory pathways, contributing to endothelial dysfunction in cardiovascular diseases). |
| 60 | 97 | **Protein tyrosine phosphatase, non-receptor type 14 (PTPN14)** (Involved in the regulation of oxidative stress and cellular signaling pathways in CVD). |
| 75 | 96 | **Chimerin 2** (Regulates redox-sensitive pathways, which influence cardiovascular disease progression, especially in vascular smooth muscle cells). |
| 82 | 95 | **RAS p21 protein activator 2 (RASA2)** (Modulates oxidative stress signaling pathways involved in endothelial cell activation and atherosclerosis). |
| 125 | 94 | **Sestrin 1** (Key regulator of oxidative stress response, involved in protecting endothelial cells from oxidative damage in cardiovascular diseases). |
| 150 | 93 | **Mitogen-activated protein kinase 7 (MAPK7)** (Regulates oxidative stress signaling pathways and vascular remodeling in CVD). |
| 506 | 80 | **Kruppel-like factor 6** (Regulates oxidative stress response by influencing antioxidant gene expression and endothelial function in CVD). |
| 515 | 80 | **Oxidation resistance 1** (Promotes resistance to oxidative damage and is involved in vascular homeostasis, playing a role in CVD). |
| 514 | 80 | **FERM domain containing 4B** (Involved in the regulation of oxidative stress pathways, which can impact endothelial cell function and CVD progression). |
| 513 | 80 | **Zinc finger protein 224** (Participates in the regulation of oxidative stress by modulating gene expression and may influence vascular diseases). |
| 509 | 80 | **Mex-3 RNA binding family member D** (Involved in the regulation of oxidative stress by modulating gene expression and vascular injury response in CVD). |
| 506 | 80 | **Kruppel-like factor 6** (Regulates oxidative stress response and has been implicated in endothelial function and atherosclerosis in CVD). |
| 510 | 80 | **Potassium voltage-gated channel subfamily D member 2** (Involved in oxidative stress regulation by modulating cellular ionic currents, which influence vascular function in CVD). |
| 515 | 80 | **Oxidation resistance 1** (Plays a key role in protecting cells from oxidative damage and is linked to vascular health in CVD). |
| 1033 | 57 | **Sirtuin 1 (SIRT1)** (SIRT1 plays a role in regulating oxidative stress by modulating cellular antioxidant responses. It also impacts the endothelial function and inflammation, both critical in CVD). |
| 1015 | 58 | **Actin, Alpha, Cardiac Muscle 1 (ACTC1)** (ACTC1 is involved in cardiac muscle contraction, and its dysfunction is associated with oxidative stress and CVD, especially in the development of heart failure). |
| 1061 | 56 | **Upstream Transcription Factor Family Member 3 (USF3)** (USF3 is linked to oxidative stress responses, and alterations in its expression are implicated in CVD, particularly through regulating genes involved in vascular dysfunction). |
| 1039 | 57 | **Cardiotrophin Like Cytokine Factor 1 (CLCF1)** (CLCF1 is involved in oxidative stress regulation and inflammation, both of which contribute to the progression of atherosclerosis and other cardiovascular pathologies). |
| 1047 | 57 | **ASXL Transcriptional Regulator 1 (ASXL1)** (ASXL1 is implicated in oxidative stress response mechanisms, and its mutations have been associated with CVD, affecting gene regulation in vascular smooth muscle cells). |
| 1100 | 55 | **Lecithin Retinol Acyltransferase (LRAT)** (LRAT regulates lipid metabolism and oxidative stress; it has been linked to atherosclerosis and CVD due to its role in managing retinol and lipid peroxidation). |
| 1145 | 53 | **Zinc Finger Protein 831 (ZNF831)** (ZNF831 is involved in antioxidant responses and has been shown to affect vascular health and the development of atherosclerotic lesions, linking it to oxidative stress and CVD). |

Top of Form

Bottom of Form

**8) Likely Targeted Genes (out of 438 predicted targets)** **by hsa-miR-200b-5p in miRDB Involved in Oxidative Stress Regulation and Cardiovascular Diseas**e

| **Target Rank** | **Target Score** | **Gene Description** |
| --- | --- | --- |
| 31 | 92 | **Thioredoxin reductase 3** (Plays a crucial role in antioxidant defense by maintaining the redox balance. Involved in regulating oxidative stress and can affect CVD via endothelial dysfunction and atherosclerosis). |
| 37 | 91 | **Peroxisome proliferator-activated receptor gamma, coactivator-related 1** (Regulates lipid metabolism and mitochondrial function, modulating oxidative stress pathways and contributing to the progression of CVD). |
| 64 | 86 | **Lysine demethylase 2B** (Involved in histone modification and regulation of gene expression, including oxidative stress responses, impacting inflammatory processes in CVD). |
| 31 | 92 | **Forkhead box C1** (Regulates antioxidant pathways and cellular stress responses, potentially influencing vascular health and CVD progression). |
| 33 | 92 | **Transcription activation suppressor family member 2** (Regulates oxidative stress response and may play a role in the pathogenesis of atherosclerosis and vascular inflammation). |

**9) Likely Targeted Genes (out of 1244 predicted targets)** **by hsa-miR-200c-3p in miRDB Involved in Oxidative Stress Regulation and Cardiovascular Diseas**e

| **Target Rank** | **Target Score** | **Gene Description** |
| --- | --- | --- |
| 1 | 100 | **Vasohibin 2** (Involved in oxidative stress regulation through angiogenesis. It also plays a role in vascular remodeling in cardiovascular disease.) |
| 2 | 100 | **Homeodomain Interacting Protein Kinase 3** (Regulates oxidative stress via its kinase activity and contributes to endothelial cell survival in CVD.) |
| 5 | 100 | **Zinc Finger E-box Binding Homeobox 1** (Regulates oxidative stress by modulating gene expression in endothelial cells, involved in atherosclerosis in CVD.) |
| 6 | 100 | **Nuclear Receptor Subfamily 5 Group A Member 2** (Modulates oxidative stress response pathways and lipid metabolism, contributing to atherosclerosis in CVD.) |
| 7 | 100 | **Zinc Finger E-box Binding Homeobox 2** (Involved in oxidative stress regulation through gene expression modulation and plays a role in vascular pathologies in CVD.) |
| 25 | 98 | **Rho GTPase Activating Protein 6** (Regulates oxidative stress signaling and is involved in endothelial dysfunction, a critical factor in CVD pathogenesis.) |
| 28 | 98 | **BRCA1 Associated Protein 1** (Modulates oxidative stress response through DNA repair mechanisms, contributing to endothelial damage in CVD.) |
| 32 | 98 | **Tripartite Motif Containing 33** (Plays a role in oxidative stress through ubiquitination processes and is implicated in endothelial dysfunction in CVD.) |
| 36 | 98 | **NOVA Alternative Splicing Regulator 2** (Regulates oxidative stress responses by modulating splicing of stress-related genes, involved in endothelial cell survival.) |
| 46 | 97 | **ELMO Domain Containing 2** (Modulates oxidative stress response in inflammatory pathways and has a role in vascular health, influencing CVD progression.) |
| 58 | 97 | **LDL Receptor Related Protein 1B** (Modulates oxidative stress through lipid metabolism and is involved in atherosclerosis development in CVD.) |
| 62 | 96 | **Family with Sequence Similarity 126 Member B** (Regulates oxidative stress through apoptotic signaling, playing a role in vascular cell death in CVD.) |
| 85 | 95 | **Very Low Density Lipoprotein Receptor** (Modulates oxidative stress pathways through lipid metabolism and is involved in atherosclerosis and CVD.) |
| 99 | 94 | **Sestrin 1** (Regulates oxidative stress and inflammation in response to stress signals, influencing vascular health and CVD progression.) |
| 513 | 80 | **Oxidation resistance 1** (Involved in protection against oxidative damage and inflammation, both key factors in CVD development.) |
| 545 | 78 | **FYN proto-oncogene, Src family tyrosine kinase** (Plays a role in cell signaling related to oxidative stress response and vascular health.) |
| 573 | 77 | **Inhibitor of nuclear factor kappa B kinase subunit beta** (Regulates NF-kB pathway, which is activated by oxidative stress and contributes to CVD pathogenesis.) |
| 604 | 76 | **Myb/SANT DNA binding domain containing 2** (Involved in transcriptional regulation of oxidative stress-related genes in cardiovascular tissue.) |
| 1031 | 57 | **Sirtuin 1** (Sirt1 regulates oxidative stress by activating antioxidant enzymes and controlling inflammation, which is crucial in the pathophysiology of CVD.) |
| 1056 | 56 | **Leucine rich repeat containing 34** (This gene has been shown to modulate oxidative stress response pathways and is implicated in endothelial dysfunction, contributing to cardiovascular diseases.) |
| 1073 | 56 | **Slingshot protein phosphatase 2** (Slingshot plays a role in regulating actin dynamics in response to oxidative stress, influencing vascular remodeling, a key feature of CVD.) |
| 1107 | 54 | **Nucleoporin 153** (It is involved in maintaining oxidative homeostasis, with implications for vascular health and the development of CVD.) |
| 1117 | 54 | **TNF receptor superfamily member 11b** (TNF receptor signaling contributes to inflammatory responses and oxidative stress, both of which are critical in the development of cardiovascular diseases.) |
| 1145 | 53 | **T cell receptor associated transmembrane adaptor 1** (This gene modulates immune responses and oxidative stress pathways that play a role in CVD, particularly in atherosclerosis and heart failure.) |
| 1150 | 53 | **Selenoprotein K** (Selenoprotein K is involved in the regulation of oxidative stress through its antioxidant properties, influencing cardiovascular health.) |
| 1163 | 53 | **ELAV like RNA binding protein 4** (ELAV-like proteins have been linked to oxidative stress regulation and contribute to inflammatory responses, which are important in CVD pathogenesis.) |

**10) Likely Targeted Genes (out of 279 predicted targets)** **by hsa-miR-200c-5p in miRDB Involved in Oxidative Stress Regulation and Cardiovascular Diseas**e

| **Target Rank** | **Target Score** | **Gene Description** |
| --- | --- | --- |
| 17 | 89 | **Collagen type XI alpha 1 chain** (Involved in vascular remodeling and fibrosis, often associated with oxidative stress and cardiovascular diseases like atherosclerosis.) |
| 18 | 89 | **Methionine sulfoxide reductase B3** (Plays a role in repairing oxidative damage in proteins, contributing to cellular survival and function in the context of CVD.) |
| 19 | 89 | **CPX chromosome region, candidate 1** (Influences cell signaling pathways and is implicated in the response to oxidative stress, particularly in the vascular system.) |
| 46 | 83 | **Mitogen-activated protein kinase 6** (Activated by oxidative stress and involved in inflammatory pathways that contribute to the progression of CVD.) |
| 47 | 83 | **Interleukin 1 receptor associated kinase 1 binding protein 1** (Plays a role in the inflammatory response and has been linked to oxidative stress in CVD pathogenesis.) |
| 61 | 80 | **Prostaglandin I2 synthase** (Involved in the regulation of vascular tone and has antioxidant properties, playing a key role in the protection against oxidative stress in cardiovascular health.) |
| 79 | 77 | **Bromodomain containing 3** (Modulates gene expression involved in oxidative stress responses and is linked to inflammatory pathways in CVD.) |

**11) Likely Targeted Genes (out of 189 predicted targets)** **by hsa-miR-128-1-5p in miRDB Involved in Oxidative Stress Regulation and Cardiovascular Diseas**e

| **Target Rank** | **Target Score** | **Gene Description** |
| --- | --- | --- |
| 19 | 78 | **Cytochrome P450 family 2 subfamily B member 6** (Involved in oxidative metabolism; plays a role in lipid metabolism and contributes to oxidative stress, influencing CVD risk) |
| 29 | 72 | **Aldehyde dehydrogenase 1 family member B1** (Participates in detoxification of reactive aldehydes, reducing oxidative stress; associated with CVD and vascular dysfunction) |
| 57 | 65 | **NADH:ubiquinone oxidoreductase core subunit S2** (Essential for mitochondrial electron transport and ATP production; dysfunction contributes to oxidative stress and cardiovascular disorders) |
| 75 | 62 | **Cytochrome b5 reductase-like** (Regulates redox reactions and oxidative stress; involved in endothelial function and CVD pathology) |
| 76 | 62 | **AKT serine/threonine kinase 2** (Modulates oxidative stress response and cell survival; dysregulation is implicated in atherosclerosis and heart disease) |
| 89 | 59 | **Synuclein gamma** (Involved in oxidative stress response; associated with endothelial dysfunction and atherosclerosis) |
| 100 | 58 | **Protein kinase C theta** (Regulates inflammatory and oxidative stress pathways; contributes to vascular dysfunction and atherosclerosis) |
| 103 | 58 | **SMAD family member 3** (Mediates oxidative stress-induced fibrosis and vascular remodeling in CVD) |
| 127 | 55 | **Methylenetetrahydrofolate reductase** (Involved in homocysteine metabolism; increased oxidative stress and CVD risk associated with its mutations) |
| 139 | 54 | **Sirtuin 6** (Protects against oxidative stress-induced damage and regulates vascular inflammation in CVD) |

| **Target Rank** | **Target Score** | **Gene Description** |
| --- | --- | --- |
| 11 | 81 | **DUSP8** (Dual specificity phosphatase 8 - Regulates oxidative stress by modulating MAPK signaling; implicated in cardiovascular pathology through vascular inflammation) |
| 18 | 78 | **CYP2B6** (Cytochrome P450 family 2 subfamily B member 6 - Involved in detoxification and oxidative stress response; contributes to CVD by influencing drug metabolism and endothelial function) |
| 20 | 76 | **FBXL5** (F-box and leucine-rich repeat protein 5 - Regulates iron homeostasis and oxidative stress, influencing atherosclerosis and cardiovascular diseases) |
| 21 | 76 | **DNAJC11** (DnaJ heat shock protein family member C11 - Mitochondrial chaperone involved in oxidative stress response; dysregulation linked to heart failure) |
| 29 | 72 | **ALDH1B1** (Aldehyde dehydrogenase 1 family member B1 - Detoxifies oxidative stress-induced aldehydes; influences cardiovascular disease by mitigating lipid peroxidation) |
| 44 | 67 | **TRAF3** (TNF receptor-associated factor 3 - Regulates inflammatory and oxidative stress pathways; contributes to vascular diseases and atherosclerosis) |
| 45 | 67 | **BACH2** (BTB domain and CNC homolog 2 - Modulates antioxidant response elements; implicated in immune regulation and cardiovascular pathology) |
| 46 | 66 | **EPHA2** (EPH receptor A2 - Influences oxidative stress in endothelial cells; associated with atherosclerosis and vascular dysfunction) |
| 56 | 65 | **NDUFS2** (NADH:ubiquinone oxidoreductase core subunit S2 - Critical for mitochondrial oxidative phosphorylation; mutations linked to oxidative stress-related cardiovascular diseases) |
| 57 | 64 | **NTN1** (Netrin 1 - Regulates oxidative stress and inflammation in vascular endothelial cells, impacting atherosclerosis progression) |

**12) Likely Targeted Genes (out of 180 predicted targets)** **by hsa-miR-128-2-5p in miRDB Involved in Oxidative Stress Regulation and Cardiovascular Diseas**e

**13) Likely Targeted Genes (out of 1254 predicted targets)** **by hsa-miR-128-3p in miRDB Involved in Oxidative Stress Regulation and Cardiovascular Diseas**e

| **Target Rank** | **Target Score** | **Gene Description** |
| --- | --- | --- |
| 29 | 97 | **Colony stimulating factor 1** (Regulates oxidative stress and inflammation, contributing to CVD pathogenesis) |
| 31 | 97 | **Nerve growth factor receptor** (Mediates oxidative stress response and vascular dysfunction in CVD) |
| 35 | 97 | **Endothelin converting enzyme 2** (Involved in vascular oxidative stress and endothelial dysfunction in CVD) |
| 62 | 96 | **Insulin receptor substrate 1** (Regulates oxidative stress and insulin signaling, impacting CVD risk) |
| 65 | 95 | **Prohibitin** (Functions in mitochondrial oxidative stress regulation and CVD development) |
| 67 | 95 | **GATA binding protein 6** (Plays a role in oxidative stress response and cardiovascular development) |
| 68 | 95 | **Adenylate kinase 2** (Involved in mitochondrial function and oxidative stress in CVD) |
| 70 | 95 | **Adenylate kinase 4** (Regulates energy metabolism and oxidative stress in cardiovascular tissues) |
| 73 | 95 | **Neogenin 1** (Mediates oxidative stress pathways affecting cardiovascular health) |
| 79 | 95 | **Transforming growth factor beta receptor 1** (Regulates oxidative stress and fibrosis in CVD) |
| 82 | 95 | **Neurofibromin 1** (Implicated in oxidative stress modulation and cardiovascular disease) |
| 83 | 95 | **Vascular endothelial growth factor C** (Critical in oxidative stress response and vascular remodeling in CVD) |
| 85 | 95 | **TLR4 interactor with leucine rich repeats** (Mediates oxidative stress and inflammatory pathways in CVD) |
| 95 | 94 | **ABL proto-oncogene 2, non-receptor tyrosine kinase** (Regulates oxidative stress and endothelial function in CVD) |
| 106 | 94 | **Mitogen-activated protein kinase 14** (Involved in oxidative stress signaling and vascular inflammation in CVD) |
| 116 | 93 | **Fibulin 5** (Plays a role in oxidative stress response and vascular elasticity in CVD) |
| 128 | 93 | **Cytochrome P450 family 39 subfamily A member 1** (Regulates oxidative stress metabolism linked to CVD) |
| 540 | 77 | **Dual specificity phosphatase 18 (DUSP18)** (Regulates oxidative stress by modulating MAPK pathways; involved in CVD by affecting vascular inflammation and endothelial function) |
| 542 | 77 | **6-Phosphofructo-2-kinase/fructose-2,6-bisphosphatase 4 (PFKFB4)** (Involved in oxidative stress response via glycolysis regulation; contributes to CVD by influencing metabolic pathways in the heart) |
| 554 | 76 | **Platelet-derived growth factor receptor alpha (PDGFRA)** (Regulates oxidative stress by modulating cellular growth and survival; plays a role in CVD by contributing to vascular remodeling and atherosclerosis) |
| 555 | 76 | **TGF-beta activated kinase 1 (MAP3K7) binding protein 3 (TAB3)** (Participates in oxidative stress responses through TGF-β signaling; associated with CVD through its role in cardiac hypertrophy and fibrosis) |
| 556 | 76 | **TNF receptor-associated factor 3 (TRAF3)** (Mediates oxidative stress response through NF-κB signaling; implicated in CVD through inflammatory and immune responses) |
| 572 | 75 | **Insulin receptor (INSR)** (Regulates oxidative stress by modulating insulin signaling and glucose metabolism; contributes to CVD by affecting endothelial function and metabolic syndrome) |
| 582 | 75 | **Galectin 3 (LGALS3)** (Plays a role in oxidative stress-induced cellular responses; involved in CVD through fibrosis and inflammation in the heart) |
| 607 | 73 | **Bone morphogenetic protein receptor type 2 (BMPR2)** (Influences oxidative stress pathways in endothelial cells; associated with pulmonary arterial hypertension and vascular dysfunction in CVD) |
| 614 | 73 | **Unc-51-like autophagy activating kinase 1 (ULK1)** (Involved in oxidative stress response via autophagy regulation; linked to CVD through its role in cardiac hypertrophy and ischemic heart disease) |
| 976 | 58 | **Caspase 1** (Involved in oxidative stress-induced inflammation and endothelial dysfunction in CVD) |
| 980 | 58 | **Vasoactive intestinal peptide** (Modulates oxidative stress and vasodilation, reducing CVD risk) |
| 986 | 58 | **Sphingomyelin phosphodiesterase 4** (Regulates oxidative stress and lipid metabolism in atherosclerosis) |
| 993 | 57 | **Apolipoprotein F** (Influences oxidative stress and lipid transport, contributing to CVD pathology) |
| 1000 | 57 | **Endothelin receptor type A** (Implicated in oxidative stress-mediated vascular dysfunction in hypertension and CVD) |
| 1015 | 57 | **Dual specificity phosphatase 5** (Regulates oxidative stress and vascular inflammation in CVD) |
| 1019 | 57 | **TNF receptor associated factor 1** (Links oxidative stress and inflammatory pathways in CVD) |
| 1054 | 56 | **Epidermal growth factor receptor (EGFR)** (Oxidative stress contributes to vascular remodeling and CVD progression) |
| 1056 | 56 | **Paraoxonase 2** (Antioxidant enzyme protecting against lipid oxidation and atherosclerosis) |
| 1072 | 55 | **Peroxisome proliferator-activated receptor gamma (PPARγ)** (Modulates oxidative stress and lipid metabolism in CVD) |
| 1078 | 55 | **Endoplasmic reticulum oxidoreductase 1 beta** (ER stress-related oxidative stress linked to endothelial dysfunction and CVD) |
| 1086 | 55 | **Interleukin 17 receptor A** (Pro-inflammatory cytokine receptor involved in oxidative stress and CVD) |
| 1088 | 55 | **FosB proto-oncogene** (AP-1 transcription factor modulating oxidative stress responses in CVD) |

**14) Likely Targeted Genes (out of 322 predicted targets)** **by hsa-miR-92a-1-5p in miRDB Involved in Oxidative Stress Regulation and Cardiovascular Diseas**e

| **Target Rank** | **Target Score** | **Gene Description** |
| --- | --- | --- |
| 1 | 98 | **SIX homeobox 4** (Regulates oxidative stress response through gene transcription modulation; implicated in cardiovascular diseases related to cellular differentiation and stress responses.) |
| 2 | 98 | **SH3 domain and tetratricopeptide repeats 2** (Involved in cell signaling and maintaining oxidative homeostasis; plays a role in endothelial dysfunction and atherosclerosis in CVD.) |
| 4 | 97 | **Neuropilin 2** (Modulates angiogenesis and vascular remodeling; implicated in oxidative stress regulation in endothelial cells, contributing to vascular complications in CVD.) |
| 6 | 95 | **Protein kinase C alpha** (PKCα regulates oxidative stress pathways, such as NADPH oxidase activation, and is involved in the development of atherosclerosis and heart failure.) |
| 7 | 95 | **Atrophin 1** (Affects oxidative stress response by modulating gene transcription related to inflammation, endothelial dysfunction, and atherosclerotic plaque formation in CVD.) |
| 17 | 90 | **C-X-C motif chemokine ligand 9** (Promotes inflammation and vascular remodeling by modulating oxidative stress in cardiovascular tissues; contributes to atherosclerotic plaque instability.) |
| 19 | 89 | **Fc fragment of IgG receptor IIIb** (Impacts oxidative stress and inflammatory response in cardiovascular diseases, contributing to immune cell activation and plaque instability in CVD.) |
| 28 | 86 | **Biogenesis of lysosomal organelles complex 1 subunit 3** (Involved in lysosomal function and autophagy, processes that regulate oxidative stress in CVD and cardiovascular cell survival.) |
| 46 | 81 | **S100 calcium binding protein A9** (Regulates oxidative stress and inflammatory response in CVD, contributing to endothelial dysfunction, and macrophage activation in atherosclerosis.) |
| 66 | 76 | **TNF superfamily member 13b** (Involved in oxidative stress and inflammation; its overexpression in cardiovascular tissues promotes plaque formation and progression in atherosclerosis.) |
| 89 | 72 | **Glutathione peroxidase 2** (Plays a direct role in neutralizing reactive oxygen species (ROS), contributing to the regulation of oxidative stress in cardiovascular tissues and protecting against endothelial injury.) |
| 96 | 71 | **Cytochrome P450 family 2 subfamily S member 1** (Involved in oxidative stress regulation through reactive oxygen species (ROS) production; contributes to vascular dysfunction in CVD.) |

**15) Likely Targeted Genes (out of 1077 predicted targets)** **by hsa-miR-92a-2-5p in miRDB Involved in Oxidative Stress Regulation and Cardiovascular Diseas**e

| **Target Rank** | **Target Score** | **Gene Description** |
| --- | --- | --- |
| 1 | 100 | **IQ motif and Sec7 domain 2** (Involved in regulating oxidative stress by modulating cellular processes related to membrane trafficking and ion transport, which impact endothelial cell function and atherosclerosis.) |
| 2 | 99 | **Forkhead box P4** (Plays a critical role in oxidative stress pathways, controlling cell survival and apoptosis, particularly in vascular smooth muscle cells in CVD.) |
| 3 | 99 | **Synaptic Ras GTPase activating protein 1** (Regulates Ras signaling, which is involved in oxidative stress responses and may contribute to the pathogenesis of CVD, particularly in vascular remodeling.) |
| 5 | 98 | **Family with sequence similarity 222 member A** (Modulates oxidative stress responses and has been implicated in cardiovascular diseases by regulating endothelial function.) |
| 7 | 97 | **Target of myb1 like 2 membrane trafficking protein** (Plays a role in maintaining cellular homeostasis under oxidative stress and contributes to endothelial dysfunction in CVD.) |
| 8 | 97 | **Hepatocyte nuclear factor 4 alpha** (Influences gene expression involved in oxidative stress regulation, impacting lipid metabolism and inflammatory pathways linked to CVD.) |
| 9 | 97 | **Sodium/potassium transporting ATPase interacting 1** (Involved in regulating oxidative stress in cardiac cells, impacting heart function and contributing to CVD.) |
| 13 | 97 | **Zinc finger and BTB domain containing 4** (Participates in oxidative stress responses and the regulation of inflammatory pathways, playing a role in the progression of CVD.) |
| 19 | 95 | **CUGBP Elav-like family member 5** (Affects oxidative stress pathways, influencing cell survival in vascular cells and contributing to the development of CVD through endothelial dysfunction.) |
| 21 | 94 | **Receptor activity modifying protein 2** (Regulates oxidative stress pathways and has been implicated in vascular smooth muscle cell behavior, contributing to CVD pathogenesis.) |
| 31 | 93 | **DnaJ heat shock protein family (Hsp40) member B5** (Plays a key role in cellular stress responses, including oxidative stress, which affects the development of CVD by modulating vascular cell survival.) |
| 35 | 93 | **Leucine rich repeats and calponin homology domain containing 4** (Involved in the regulation of oxidative stress, and its expression impacts vascular remodeling associated with CVD.) |
| 42 | 92 | **Beta-1,4-N-acetyl-galactosaminyltransferase 1** (Plays a role in glycosylation processes, influencing oxidative stress regulation in vascular endothelial cells, thus impacting CVD.) |
| 54 | 92 | **Pappalysin 2** (Involved in oxidative stress regulation in the heart and may influence the progression of CVD by modulating extracellular matrix remodeling and fibrosis.) |
| 70 | 90 | **UDP glycosyltransferase family 3 member A1** (Plays a role in oxidative stress regulation by modulating lipid metabolism and inflammation pathways, contributing to CVD.) |
| 76 | 89 | **ZFP36 ring finger protein like 1** (A critical regulator of inflammation and oxidative stress, contributing to vascular inflammation and the development of atherosclerosis and CVD.) |
| 91 | 88 | **Mitochondrial antiviral signaling protein** (Regulates oxidative stress and inflammation pathways, influencing CVD through modulation of mitochondrial function in cardiac cells.) |
| 105 | 87 | **Lysine methyltransferase 2D** (Modulates oxidative stress by influencing epigenetic regulation in cardiovascular cells, contributing to vascular remodeling in CVD.) |
| 124 | 86 | **SWI/SNF related, matrix associated, actin dependent regulator of chromatin subfamily c member 2** (Influences gene expression in response to oxidative stress, impacting vascular function in CVD.) |
| 135 | 86 | **Methyl-CpG binding domain protein 3 like 3** (Involved in oxidative stress response and vascular smooth muscle cell function, playing a role in the pathogenesis of CVD.) |
| 149 | 85 | **Spi-B transcription factor** (Modulates oxidative stress responses in immune cells and endothelial cells, contributing to vascular dysfunction and CVD.) |
| 509 | 65 | **CD276 molecule** (Involved in inflammation and oxidative stress by modulating immune responses; its upregulation is linked to CVD through immune dysregulation.) |
| 510 | 65 | **Phospholipase A2 group XV** (Regulates the release of arachidonic acid and contributes to inflammation and oxidative stress in CVD and other inflammatory conditions.) |
| 544 | 64 | **TIMP metallopeptidase inhibitor 3** (Inhibits matrix metalloproteinases, which are involved in oxidative stress and ECM degradation in CVD.) |
| 579 | 63 | **Angiopoietin 2** (Plays a role in endothelial dysfunction and inflammation, which are central to oxidative stress and CVD development.) |
| 588 | 63 | **Extracellular matrix protein 1** (Contributes to vascular remodeling and response to oxidative stress in CVD, influencing fibrotic responses.) |
| 658 | 60 | **Nuclear factor kappa B subunit 2** (Central in regulating inflammation and oxidative stress, with key involvement in CVD pathogenesis through inflammatory pathways.) |
| 826 | 56 | **Potassium channel tetramerization domain containing 17** (**KCNK17**) (Plays a role in cell membrane potential regulation and oxidative stress response, influencing cardiovascular function) |
| 827 | 56 | **ATPase phospholipid transporting 11A** (**ATP11A**) (Regulates cellular lipid homeostasis, affecting oxidative stress and inflammation, contributing to CVD progression) |
| 828 | 56 | **Apelin receptor** (**APLNR**) (Involved in vasodilation and inflammation, modulating oxidative stress in cardiovascular tissues) |
| 829 | 56 | **Plexin A4** (**PLXNA4**) (A receptor for semaphorins, modulates oxidative stress, and has been implicated in cardiovascular remodeling) |
| 830 | 56 | **Speckle type BTB/POZ protein** (**SPOP**) (Regulates oxidative stress response and has a role in cell survival, influencing cardiovascular pathology) |
| 831 | 56 | **FYVE and coiled-coil domain containing 1** (**FYCO1**) (Involved in cellular stress response, including oxidative stress and cellular homeostasis, linked to CVD) |
| 832 | 56 | **Dynein light chain roadblock-type 1** (**DYNLRB1**) (Plays a role in cellular transport, linked to oxidative stress and vascular health) |
| 833 | 56 | **Ring finger and FYVE like domain containing E3 ubiquitin protein ligase** (**RNF183**) (Regulates oxidative stress via protein ubiquitination, potentially contributing to cardiovascular disease) |
| 834 | 56 | **Cytochrome c oxidase assembly factor heme A:farnesyltransferase COX10** (**COX10**) (Crucial for mitochondrial function and energy production, regulating oxidative stress in cardiac tissues) |
| 835 | 56 | **OPA3, outer mitochondrial membrane lipid metabolism regulator** (**OPA3**) (Affects mitochondrial lipid metabolism, with implications for oxidative stress in CVD) |
| 836 | 56 | **Purinergic receptor P2Y2** (**P2RY2**) (Regulates cell signaling and oxidative stress, potentially affecting vascular function and CVD) |
| 837 | 56 | **Chromosome 16 open reading frame 58** (**C16orf58**) (A gene that may play a role in oxidative stress modulation and has been associated with cardiovascular health) |
| 838 | 56 | **Tensin 4** (**TNS4**) (Involved in focal adhesion and cell signaling, which are modulated by oxidative stress in CVD) |
| 839 | 56 | **FLT3 interacting zinc finger 1** (**FIZ1**) (Regulates cell proliferation and survival in oxidative stress conditions, influencing cardiovascular outcomes) |
| 840 | 56 | **MYC associated zinc finger protein** (**MAZ**) (Involved in gene transcription regulation under oxidative stress, contributing to cardiovascular disease) |
| 841 | 56 | **Tubulointerstitial nephritis antigen like 1** (**TINAGL1**) (Regulates oxidative stress response, with potential effects on kidney function in CVD) |
| 842 | 56 | **Serine protease 53** (**PRSS53**) (Involved in protein cleavage and stress response, linked to oxidative stress in cardiovascular diseases) |
| 843 | 56 | **IQ motif containing E** (**IQCE**) (Regulates cellular calcium levels and oxidative stress, influencing vascular health) |
| 844 | 56 | **Gamma-aminobutyric acid type A receptor beta2 subunit** (**GABRB2**) (Regulates GABAergic neurotransmission, modulating oxidative stress responses in the cardiovascular system) |
| 845 | 56 | **Phosphate cytidylyltransferase 2, ethanolamine** (**PCYT2**) (Involved in lipid biosynthesis and oxidative stress regulation, potentially affecting CVD) |
| 846 | 56 | **Src kinase associated phosphoprotein 1** (**SKAP1**) (Modulates cell signaling pathways, contributing to oxidative stress in cardiovascular disease) |
| 847 | 55 | **RAB18, member RAS oncogene family** (**RAB18**) (Involved in vesicle trafficking and oxidative stress response, influencing cardiovascular health) |
| 848 | 55 | **Family with sequence similarity 129 member C** (**FAM129C**) (Regulates stress responses, including oxidative stress, with potential relevance in CVD) |
| 849 | 55 | **Ataxin 7 like 1** (**ATXN7L1**) (Plays a role in cellular stress responses, with implications for oxidative stress and cardiovascular disease) |
| 850 | 55 | **Phosphatidylinositol-5-phosphate 4-kinase type 2 beta** (**PIP4K2B**) (Regulates phosphoinositide metabolism and cellular stress, with links to cardiovascular disease) |

**16) Likely Targeted Genes (out of 985 predicted targets)** **by hsa-miR-92a-3p in miRDB Involved in Oxidative Stress Regulation and Cardiovascular Diseas**e

| **Target Rank** | **Target Score** | **Gene Description (Role in Oxidative Stress and CVD)** |
| --- | --- | --- |
| 41 | 97 | **NADPH oxidase 4** (Plays a key role in generating reactive oxygen species, contributing to oxidative stress in CVD, and promoting endothelial dysfunction and atherosclerosis.) |
| 39 | 97 | **Sestrin 3** (Protects against oxidative stress by regulating reactive oxygen species and inflammation, playing a protective role in cardiovascular health.) |
| 10 | 99 | **Mitogen-activated protein kinase kinase 4** (Involved in stress-induced signaling pathways, affecting cell survival and apoptosis, contributing to cardiovascular damage during oxidative stress.) |
| 41 | 97 | **NADPH oxidase 4** (A major contributor to the production of superoxide anion in endothelial cells, playing a central role in the progression of oxidative stress in cardiovascular diseases.) |
| 491 | 79 | **Roundabout guidance receptor 1** (Regulates angiogenesis and vascular remodeling, involved in oxidative stress pathways) |
| 492 | 79 | **ATPase plasma membrane Ca2+ transporting 4** (Regulates calcium homeostasis, affecting oxidative stress response and vascular function in CVD) |
| 493 | 79 | **KIF1 binding protein** (Associated with mitochondrial dynamics, which plays a role in oxidative stress responses and heart function) |
| 494 | 79 | **Solute carrier family 52 member 1** (Involved in cellular transport and oxidative stress regulation, may impact vascular health in CVD) |
| 495 | 79 | **Integral membrane protein 2B** (Involved in cellular stress responses, particularly in vascular smooth muscle cells related to CVD) |
| 496 | 79 | **Kinesin family member 5B** (Impacts mitochondrial function, a critical component in oxidative stress regulation and CVD) |
| 497 | 79 | **DNA polymerase kappa** (Plays a role in DNA repair, important for mitigating oxidative damage in CVD and heart cell function) |
| 498 | 79 | **PPARG coactivator 1 beta** (Regulates mitochondrial function and oxidative stress, crucial in vascular inflammation in CVD) |
| 499 | 79 | **Serum/glucocorticoid regulated kinase 1** (Involved in cellular responses to oxidative stress, contributing to CVD development) |
| 500 | 79 | **SPARC (osteonectin), cwcv and kazal like domains proteoglycan 2** (Involved in extracellular matrix remodeling and oxidative stress response in CVD) |
| 501 | 78 | **Trafficking kinesin protein 2** (Regulates mitochondrial trafficking and oxidative stress, potentially affecting heart function in CVD) |
| 502 | 78 | **PDS5 cohesin associated factor B** (Influences DNA repair and oxidative stress responses, contributing to vascular health in CVD) |
| 503 | 78 | **Antizyme inhibitor 1** (Regulates polyamine metabolism, which can modulate oxidative stress in cardiovascular tissue) |
| 504 | 78 | **Protein kinase C epsilon** (Involved in signaling pathways that regulate oxidative stress, affecting vascular function and CVD) |
| 505 | 78 | **Choline kinase alpha** (Plays a role in phospholipid metabolism, affecting oxidative stress responses and heart function in CVD) |
| 506 | 78 | **ATP6V1F neighbor** (Regulates intracellular acidification, a process linked to oxidative stress and endothelial function in CVD) |
| 507 | 78 | **Fibronectin type III domain containing 3B** (Modulates extracellular matrix components, potentially influencing oxidative stress in CVD) |
| 508 | 78 | **Double PHD fingers 3** (Involved in protein interactions and stress responses, impacting vascular health and oxidative damage in CVD) |
| 509 | 78 | **Integrin binding sialoprotein** (Regulates cell signaling and oxidative stress, important in vascular smooth muscle cells in CVD) |
| 510 | 78 | **Tetratricopeptide repeat domain 17** (Plays a role in protein folding and oxidative stress regulation, influencing CVD outcomes) |
